# Bis[3,3′-(piperazine-1,4-di­yl)­dipropan­aminium] di-μ_2_-sulfido-bis­[disulfido­german­ate(IV)]

**DOI:** 10.1107/S1600536812030334

**Published:** 2012-07-10

**Authors:** Nian-Nian Wang, Chao Xu, Taike Duan, Qun Chen, Qian-Feng Zhang

**Affiliations:** a Institute of Molecular Engineering and Applied Chemistry, Anhui University of Technology, Ma’anshan, Anhui 243002, People’s Republic of China; b Department of Applied Chemistry, School of Petrochemical Engineering, Changzhou University, Jiangsu 213164, People’s Republic of China

## Abstract

In the title compound, (C_10_H_26_N_4_)_2_[Ge_2_S_6_], the dimeric [Ge_2_S_6_]^4−^ anion formed by two edge-sharing GeS_4_ tetra­hedral units lies around an inversion centre. The average terminal and bridging Ge—S bond lengths are 2.162 (7) and 2.267 (15) Å, respectively. The inorganic anions and organic cations are organized into a three-dimensional network by numerous N—H⋯S hydrogen bonds.

## Related literature

For background to main group metal–chalcogenide compounds, see: Bedard *et al.* (1999 ▶); Nellis *et al.* (1995 ▶); Blachnik & Fehlker (2001 ▶); Zheng *et al.* (2002 ▶, 2005 ▶). For related structures, see: Jia *et al.* (2005 ▶); Xu *et al.* (2012 ▶).
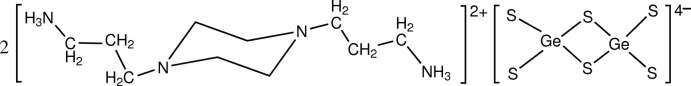


## Experimental

### 

#### Crystal data

(C_10_H_26_N_4_)_2_[Ge_2_S_6_]
*M* *_r_* = 742.24
Monoclinic, 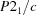

*a* = 12.0111 (4) Å
*b* = 7.7759 (3) Å
*c* = 18.9777 (7) Å
β = 103.569 (1)°
*V* = 1722.99 (11) Å^3^
*Z* = 2
Mo *K*α radiation
μ = 2.13 mm^−1^
*T* = 296 K
0.38 × 0.29 × 0.16 mm

#### Data collection

Bruker APEXII CCD area-detector diffractometer
Absorption correction: multi-scan (*SADABS*; Sheldrick, 1997 ▶) *T*_min_ = 0.498, *T*_max_ = 0.727
16480 measured reflections
3950 independent reflections
3194 reflections with *I* > 2σ(*I*)
*R*_int_ = 0.021

#### Refinement

*R*[*F* ^2^ > 2σ(*F* ^2^)] = 0.039
*wR*(*F* ^2^) = 0.113
*S* = 1.07
3950 reflections
165 parameters
H-atom parameters constrained
Δρ_max_ = 1.15 e Å^−3^
Δρ_min_ = −0.37 e Å^−3^

### 

Data collection: *APEX2* (Bruker, 2005 ▶); cell refinement: *SAINT* (Bruker, 2005 ▶); data reduction: *SAINT*; program(s) used to solve structure: *SHELXS97* (Sheldrick, 2008 ▶); program(s) used to refine structure: *SHELXL97* (Sheldrick, 2008 ▶); molecular graphics: *SHELXTL* (Sheldrick, 2008 ▶); software used to prepare material for publication: *SHELXTL*.

## Supplementary Material

Crystal structure: contains datablock(s) I, global. DOI: 10.1107/S1600536812030334/gk2485sup1.cif

Structure factors: contains datablock(s) I. DOI: 10.1107/S1600536812030334/gk2485Isup2.hkl

Additional supplementary materials:  crystallographic information; 3D view; checkCIF report

## Figures and Tables

**Table 1:** Hydrogen-bond geometry (Å, °)

*D*—H⋯*A*	*D*—H	H⋯*A*	*D*⋯*A*	*D*—H⋯*A*
N3—H3*B*⋯S2^i^	0.89	2.48	3.278 (3)	149
N3—H3*C*⋯S3	0.89	2.34	3.225 (3)	170
N3—H3*A*⋯S2^ii^	0.89	2.61	3.440 (3)	157
N3—H3*A*⋯S3^ii^	0.89	2.83	3.366 (3)	120
N4—H4*C*⋯S2^iii^	0.89	2.53	3.408 (4)	169
N4—H4*B*⋯S2^iv^	0.89	2.34	3.228 (4)	178
N4—H4*A*⋯S3^v^	0.89	2.39	3.275 (4)	173

Symmetry codes: (i) 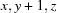; (ii) 
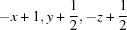
; (iii) 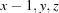; (iv) 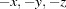; (v) 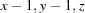.

**Fig. 1.**
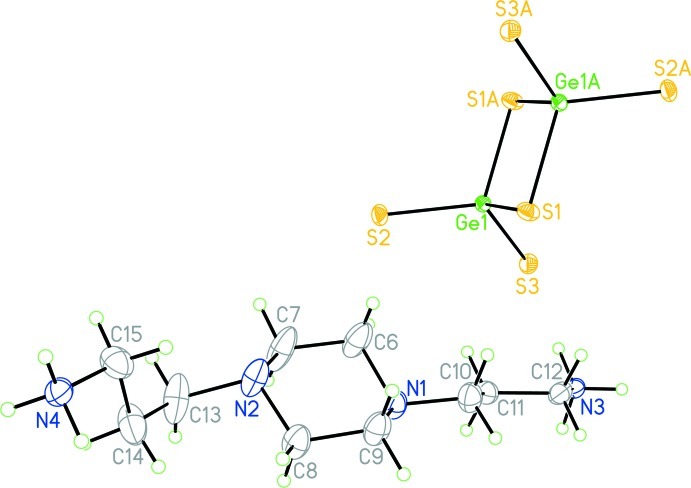
The structure of the title compound, showing displacement ellipsoids at the 50% probability level. Atoms with the A label were generated by the symmetry operation -x+1, -y+1, -z.

**Fig. 2.**
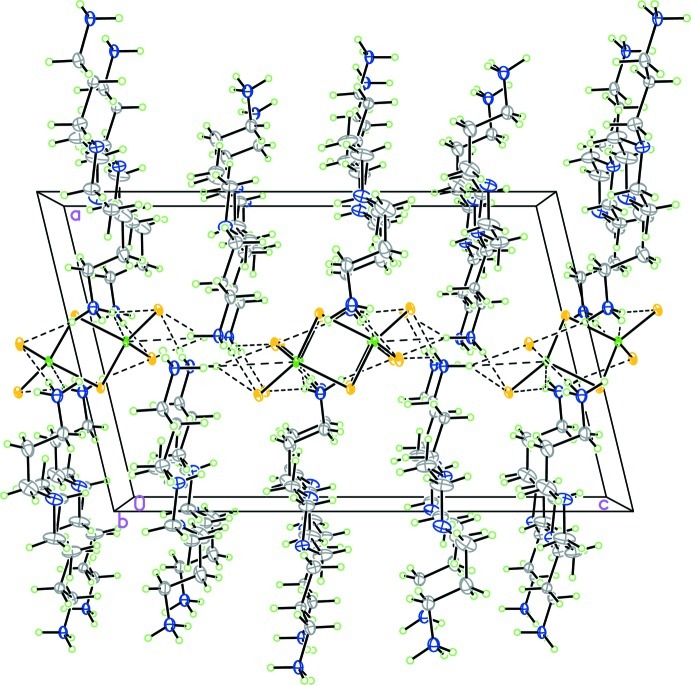
Packing diagram of the title compound. Dashed lines donote hydrogen bonds.

## supplementary crystallographic information

### Comment

Since Bedard reported the first porous metal chalcogenide open framework in 1999 (Bedard *et al.*, 1999), a series of binary and ternary metal chalcogenide open-frameworks have been synthesized (Nellis *et al.*, 1995; Zheng *et al.*, 2005). Among the various synthetic methods, hydrothermal technique is the best choice for preparing related compounds due to gentle reaction conditions. Moreover, organic amines are often used as templates in the hydrothermal reactions. Therefore, amines with different structures play an important role for templating effect in the construction of open-frameworks (Zheng *et al.*, 2002). In this paper, we report the hydrothermal synthesis and crystal structure of an amine-templated thiogermanate, [bappH_2_]_2_[Ge_2_S_6_] (bapp = 1,4-bis(3-aminopropyl)piperazine).

The title compound crystallizes in the monoclinic space group *P*2_1_/*c* with a dimeric anion of [Ge_2_S_6_]^4- ^located around inversion centre and with diprotonated 1,4-bis(3-aminopropyl)- piperazine in general position (Fig. 1). The dimeric [Ge_2_S_6_]^4-^ anion is constructed by two edge-sharing tetrahedral GeS_4_ units forming a planar Ge_2_S_2_ quadrilateral with the four terminal sulfur atoms lying on a perpendicular plane. The S—Ge—S angles in tetrahedral GeS_4_ unit are in the ranges from 93.82 (3) to 114.12 (4)°. The average bond length of Ge—S_t_ (terminal bond) of 2.162 (7) Å is obviously shorter than that of Ge—S_b_ (bridging bond) [2.267 (15) Å]. The bond length values are similar to those found in the other thiogermanates (Jia *et al.* 2005; Xu *et al.*, 2012). The two terminal amine groups of 4-bis(3-aminopropyl)piperazine are protonated to balance negative charges of the dimeric anion. The [Ge_2_S_6_]^4-^ anions and [bappH_2_]^2+^ cations are organized into an extended three-dimensional network by N—H···S hydrogen bonds (Fig. 2 and Table 1).

### Experimental

GeO_2_ (104.6 mg, 1.0 mmol) and S powder (128.0 mg, 4.0 mmol) in the distilled water (4.8550 g) were mixed with 1,4-bis(3-aminopropyl)piperazine (2.5640 g) in a 23 mL Teflon-lined stainless steel autoclave to and stirred for 20 min. The vessel was sealed and heated to 190°C for 6 d and then cooled to room temperature. Colorless flake crystals were obtained and air dried. The yield based on GeO_2_ is about 45%. Analysis, calculated for C_20_H_52_N_8_S_6_Ge_2_: C 32.4, H 7.06, N 15.1%; found C 32.2, H 6.98, N 14.8 %.

### Refinement

All C-bound H atoms were positioned geometrically and refined as riding atoms with C—H = 0.97 Å and *U*_iso_(H) = 1.2*U*_eq_(C)]. N-bound H atoms were located from a difference Fourier map but for final refinement they were were positioned geometrically with N—H = 0.89 Å and *U*_iso_(H) = 1.5*U*_eq_(N)].

### Crystal data

**Table:**

(C_10_H_26_N_4_)_2_[Ge_2_S_6_]	*F*(000) = 776
*M**_r_* = 742.24	*D*_x_ = 1.431 Mg m^−^^3^
Monoclinic, *P*2_1_/*c*	Mo *K*α radiation, λ = 0.71073 Å
Hall symbol: -P 2ybc	Cell parameters from 6783 reflections
*a* = 12.0111 (4) Å	θ = 2.5–27.0°
*b* = 7.7759 (3) Å	µ = 2.13 mm^−^^1^
*c* = 18.9777 (7) Å	*T* = 296 K
β = 103.569 (1)°	Block, colourless
*V* = 1722.99 (11) Å^3^	0.38 × 0.29 × 0.16 mm
*Z* = 2	

### Data collection

**Table:**

Bruker APEXII CCD area-detector diffractometer	3950 independent reflections
Radiation source: fine-focus sealed tube	3194 reflections with *I* > 2σ(*I*)
Graphite monochromator	*R*_int_ = 0.021
phi and ω scans	θ_max_ = 27.5°, θ_min_ = 1.7°
Absorption correction: multi-scan (*SADABS*; Sheldrick, 1997)	*h* = −15→15
*T*_min_ = 0.498, *T*_max_ = 0.727	*k* = −10→6
16480 measured reflections	*l* = −22→24

### Refinement

**Table:**

Refinement on *F*^2^	Primary atom site location: structure-invariant direct methods
Least-squares matrix: full	Secondary atom site location: difference Fourier map
*R*[*F*^2^ > 2σ(*F*^2^)] = 0.039	Hydrogen site location: inferred from neighbouring sites
*wR*(*F*^2^) = 0.113	H-atom parameters constrained
*S* = 1.07	*w* = 1/[σ^2^(*F*_o_^2^) + (0.0586*P*)^2^ + 1.3201*P*] where *P* = (*F*_o_^2^ + 2*F*_c_^2^)/3
3950 reflections	(Δ/σ)_max_ = 0.001
165 parameters	Δρ_max_ = 1.15 e Å^−^^3^
0 restraints	Δρ_min_ = −0.37 e Å^−^^3^

### Special details

**Table:**

Geometry. All esds (except the esd in the dihedral angle between two l.s. planes) are estimated using the full covariance matrix. The cell esds are taken into account individually in the estimation of esds in distances, angles and torsion angles; correlations between esds in cell parameters are only used when they are defined by crystal symmetry. An approximate (isotropic) treatment of cell esds is used for estimating esds involving l.s. planes.
Refinement. Refinement of F^2^ against ALL reflections. The weighted R-factor wR and goodness of fit S are based on F^2^, conventional R-factors R are based on F, with F set to zero for negative F^2^. The threshold expression of F^2^ > 2sigma(F^2^) is used only for calculating R-factors(gt) etc. and is not relevant to the choice of reflections for refinement. R-factors based on F^2^ are statistically about twice as large as those based on F, and R- factors based on ALL data will be even larger.

### Fractional atomic coordinates and isotropic or equivalent isotropic displacement parameters (Å^2^)

**Table:**

	*x*	*y*	*z*	*U*_iso_*/*U*_eq_	
Ge1	0.53261 (3)	0.44178 (4)	0.080248 (14)	0.04128 (12)	
S1	0.38171 (8)	0.59109 (12)	0.01275 (4)	0.0541 (2)	
S2	0.47606 (9)	0.19876 (11)	0.11556 (4)	0.0606 (3)	
S3	0.63552 (8)	0.59389 (11)	0.16645 (4)	0.0565 (2)	
N1	0.1088 (3)	0.4905 (5)	0.1532 (2)	0.0769 (10)	
N2	−0.0300 (3)	0.1852 (5)	0.1210 (3)	0.1023 (15)	
N3	0.4526 (3)	0.8528 (4)	0.20970 (15)	0.0630 (8)	
H3A	0.4580	0.8401	0.2570	0.094*	
H3B	0.4769	0.9572	0.2013	0.094*	
H3C	0.4956	0.7738	0.1948	0.094*	
N4	−0.3627 (4)	−0.0949 (5)	0.0505 (2)	0.0782 (10)	
H4A	−0.3673	−0.1851	0.0785	0.117*	
H4B	−0.3923	−0.1222	0.0043	0.117*	
H4C	−0.4016	−0.0072	0.0630	0.117*	
C6	0.1475 (5)	0.3408 (7)	0.1201 (5)	0.121 (2)	
H6A	0.2301	0.3319	0.1362	0.145*	
H6B	0.1284	0.3547	0.0679	0.145*	
C7	0.0954 (5)	0.1827 (7)	0.1388 (5)	0.146 (3)	
H7A	0.1227	0.1621	0.1903	0.175*	
H7B	0.1207	0.0875	0.1134	0.175*	
C8	−0.0710 (4)	0.3401 (7)	0.1474 (4)	0.0981 (16)	
H8A	−0.0559	0.3348	0.1999	0.118*	
H8B	−0.1532	0.3475	0.1288	0.118*	
C9	−0.0168 (4)	0.4974 (6)	0.1262 (4)	0.0932 (15)	
H9A	−0.0357	0.5077	0.0738	0.112*	
H9B	−0.0466	0.5979	0.1459	0.112*	
C10	0.1646 (4)	0.6473 (6)	0.1389 (3)	0.0880 (13)	
H10A	0.1219	0.7451	0.1504	0.106*	
H10B	0.1631	0.6528	0.0876	0.106*	
C11	0.2881 (4)	0.6607 (6)	0.1822 (2)	0.0713 (10)	
H11A	0.2915	0.6441	0.2334	0.086*	
H4N	0.3338	0.5716	0.1669	0.086*	
C12	0.3349 (4)	0.8321 (5)	0.1708 (2)	0.0725 (11)	
H12A	0.3294	0.8485	0.1195	0.087*	
H12B	0.2888	0.9201	0.1865	0.087*	
C13	−0.0732 (7)	0.0324 (9)	0.1558 (5)	0.151 (3)	
H13A	−0.0359	−0.0697	0.1430	0.182*	
H13B	−0.0489	0.0458	0.2079	0.182*	
C14	−0.1959 (7)	0.0022 (10)	0.1373 (3)	0.129 (3)	
H14A	−0.2341	0.1056	0.1479	0.155*	
H14B	−0.2130	−0.0889	0.1680	0.155*	
C15	−0.2442 (5)	−0.0463 (8)	0.0594 (3)	0.1042 (18)	
H15A	−0.2385	0.0503	0.0281	0.125*	
H15B	−0.2012	−0.1416	0.0461	0.125*	

### Atomic displacement parameters (Å^2^)

**Table:**

	*U*^11^	*U*^22^	*U*^33^	*U*^12^	*U*^13^	*U*^23^
Ge1	0.0602 (2)	0.03395 (18)	0.02960 (17)	0.00094 (13)	0.01036 (13)	0.00344 (11)
S1	0.0626 (5)	0.0634 (5)	0.0388 (4)	0.0157 (4)	0.0166 (3)	0.0086 (3)
S2	0.0973 (7)	0.0428 (4)	0.0406 (4)	−0.0147 (4)	0.0140 (4)	0.0072 (3)
S3	0.0768 (6)	0.0487 (5)	0.0396 (4)	−0.0091 (4)	0.0046 (4)	−0.0021 (3)
N1	0.066 (2)	0.0546 (18)	0.111 (3)	0.0014 (16)	0.0225 (19)	−0.0006 (19)
N2	0.076 (2)	0.062 (2)	0.152 (4)	−0.0009 (19)	−0.008 (3)	−0.010 (3)
N3	0.099 (2)	0.0472 (16)	0.0448 (15)	−0.0004 (16)	0.0211 (15)	−0.0012 (12)
N4	0.102 (3)	0.068 (2)	0.066 (2)	−0.0073 (19)	0.0226 (19)	−0.0058 (17)
C6	0.077 (3)	0.074 (3)	0.216 (7)	0.006 (3)	0.040 (4)	−0.035 (4)
C7	0.078 (3)	0.062 (3)	0.271 (10)	0.010 (3)	−0.014 (5)	−0.027 (4)
C8	0.068 (3)	0.083 (3)	0.138 (5)	−0.008 (2)	0.012 (3)	−0.011 (3)
C9	0.073 (3)	0.066 (3)	0.136 (5)	0.013 (2)	0.014 (3)	0.002 (3)
C10	0.088 (3)	0.068 (3)	0.109 (4)	0.005 (2)	0.026 (3)	0.013 (3)
C11	0.074 (2)	0.075 (3)	0.066 (2)	0.000 (2)	0.0193 (19)	−0.001 (2)
C12	0.101 (3)	0.058 (2)	0.056 (2)	0.014 (2)	0.014 (2)	−0.0031 (18)
C13	0.147 (6)	0.091 (4)	0.185 (8)	−0.047 (4)	−0.022 (6)	0.026 (5)
C14	0.157 (6)	0.136 (5)	0.086 (4)	−0.068 (5)	0.012 (4)	0.015 (4)
C15	0.107 (4)	0.128 (5)	0.079 (3)	−0.018 (3)	0.025 (3)	0.004 (3)

### Geometric parameters (Å, º)

**Table:**

Ge1—S3	2.1573 (9)	C7—H7B	0.9700
Ge1—S2	2.1669 (9)	C8—C9	1.485 (7)
Ge1—S1	2.2770 (9)	C8—H8A	0.9700
S1—Ge1^i^	2.2564 (8)	C8—H8B	0.9700
N1—C10	1.448 (6)	C9—H9A	0.9700
N1—C6	1.450 (6)	C9—H9B	0.9700
N1—C9	1.476 (6)	C10—C11	1.522 (6)
N2—C8	1.435 (7)	C10—H10A	0.9700
N2—C7	1.465 (7)	C10—H10B	0.9700
N2—C13	1.509 (8)	C11—C12	1.482 (6)
N3—C12	1.442 (5)	C11—H11A	0.9700
N3—H3A	0.8900	C11—H4N	0.9700
N3—H3B	0.8900	C12—H12A	0.9700
N3—H3C	0.8900	C12—H12B	0.9700
N4—C15	1.443 (7)	C13—C14	1.451 (10)
N4—H4A	0.8900	C13—H13A	0.9700
N4—H4B	0.8900	C13—H13B	0.9700
N4—H4C	0.8900	C14—C15	1.504 (8)
C6—C7	1.460 (8)	C14—H14A	0.9700
C6—H6A	0.9700	C14—H14B	0.9700
C6—H6B	0.9700	C15—H15A	0.9700
C7—H7A	0.9700	C15—H15B	0.9700
			
S3—Ge1—S2	114.15 (3)	N1—C9—C8	110.6 (4)
S3—Ge1—S1^i^	111.71 (4)	N1—C9—H9A	109.5
S2—Ge1—S1^i^	112.13 (4)	C8—C9—H9A	109.5
S3—Ge1—S1	112.70 (4)	N1—C9—H9B	109.5
S2—Ge1—S1	110.66 (4)	C8—C9—H9B	109.5
S1^i^—Ge1—S1	93.82 (3)	H9A—C9—H9B	108.1
Ge1^i^—S1—Ge1	86.18 (3)	N1—C10—C11	113.1 (4)
C10—N1—C6	112.7 (4)	N1—C10—H10A	109.0
C10—N1—C9	112.6 (4)	C11—C10—H10A	109.0
C6—N1—C9	106.5 (4)	N1—C10—H10B	109.0
C8—N2—C7	110.4 (4)	C11—C10—H10B	109.0
C8—N2—C13	109.1 (6)	H10A—C10—H10B	107.8
C7—N2—C13	109.2 (5)	C12—C11—C10	109.7 (4)
C12—N3—H3A	109.5	C12—C11—H11A	109.7
C12—N3—H3B	109.5	C10—C11—H11A	109.7
H3A—N3—H3B	109.5	C12—C11—H4N	109.7
C12—N3—H3C	109.5	C10—C11—H4N	109.7
H3A—N3—H3C	109.5	H11A—C11—H4N	108.2
H3B—N3—H3C	109.5	N3—C12—C11	112.7 (3)
C15—N4—H4A	109.5	N3—C12—H12A	109.1
C15—N4—H4B	109.5	C11—C12—H12A	109.1
H4A—N4—H4B	109.5	N3—C12—H12B	109.1
C15—N4—H4C	109.5	C11—C12—H12B	109.1
H4A—N4—H4C	109.5	H12A—C12—H12B	107.8
H4B—N4—H4C	109.5	C14—C13—N2	117.1 (6)
N1—C6—C7	111.8 (5)	C14—C13—H13A	108.0
N1—C6—H6A	109.3	N2—C13—H13A	108.0
C7—C6—H6A	109.3	C14—C13—H13B	108.0
N1—C6—H6B	109.3	N2—C13—H13B	108.0
C7—C6—H6B	109.3	H13A—C13—H13B	107.3
H6A—C6—H6B	107.9	C13—C14—C15	114.5 (7)
C6—C7—N2	114.2 (5)	C13—C14—H14A	108.6
C6—C7—H7A	108.7	C15—C14—H14A	108.6
N2—C7—H7A	108.7	C13—C14—H14B	108.6
C6—C7—H7B	108.7	C15—C14—H14B	108.6
N2—C7—H7B	108.7	H14A—C14—H14B	107.6
H7A—C7—H7B	107.6	N4—C15—C14	108.9 (5)
N2—C8—C9	112.9 (5)	N4—C15—H15A	109.9
N2—C8—H8A	109.0	C14—C15—H15A	109.9
C9—C8—H8A	109.0	N4—C15—H15B	109.9
N2—C8—H8B	109.0	C14—C15—H15B	109.9
C9—C8—H8B	109.0	H15A—C15—H15B	108.3
H8A—C8—H8B	107.8		

Symmetry code: (i) −*x*+1, −*y*+1, −*z*.

### Hydrogen-bond geometry (Å, º)

**Table:**

*D*—H···*A*	*D*—H	H···*A*	*D*···*A*	*D*—H···*A*
N3—H3*B*···S2^ii^	0.89	2.48	3.278 (3)	149
N3—H3*C*···S3	0.89	2.34	3.225 (3)	170
N3—H3*A*···S2^iii^	0.89	2.61	3.440 (3)	157
N3—H3*A*···S3^iii^	0.89	2.83	3.366 (3)	120
N4—H4*C*···S2^iv^	0.89	2.53	3.408 (4)	169
N4—H4*B*···S2^v^	0.89	2.34	3.228 (4)	178
N4—H4*A*···S3^vi^	0.89	2.39	3.275 (4)	173

Symmetry codes: (ii) *x*, *y*+1, *z*; (iii) −*x*+1, *y*+1/2, −*z*+1/2; (iv) *x*−1, *y*, *z*; (v) −*x*, −*y*, −*z*; (vi) *x*−1, *y*−1, *z*.
